# A novel vision-based system for quantitative analysis of abdominal aortic aneurysm deformation

**DOI:** 10.1186/s12938-019-0681-y

**Published:** 2019-05-14

**Authors:** Andrzej Polanczyk, Michal Podgorski, Maciej Polanczyk, Aleksandra Piechota-Polanczyk, Ludomir Stefanczyk, Michal Strzelecki

**Affiliations:** 1 Faculty of Process and Environmental Engineering, Department of Heat and Mass Transfer, Lodz University of Technology, Łódź, Poland; 2 Department of Radiology and Diagnostic Imaging, Medical University of Lodz, Łódź, Poland; 3 Institute of Electronics, Lodz University of Technology, Łódź, Poland; 4 Department of Medical Biotechnology, Jagiellonian University, Kraków, Poland

**Keywords:** Non-contact strain measurement, Digital image correlation, Deformation measurement, Displacement measurement, Optical strain, Strain distribution testing

## Abstract

**Background:**

In clinical diagnostics, combination of different imaging techniques is applied to assess spatial configuration of the abdominal aortic aneurysm (AAA) and deformation of its wall. As deformation of aneurysm wall is crucial parameter in assessing wall rupture, we aimed to develop and validate a Non-Invasive Vision-Based System (NIVBS) for the analysis of 3D elastic artificial abdominal aortic models. 3D-printed elastic AAA models from four patients were applied for the reconstruction of real hemodynamic. During experiments, the inlet boundary conditions included the injection volume and frequency of pulsation averaged from electrocardiography traces. NIVBS system was equipped with nine cameras placed at a constant distance to record wall movement from 360^o^ angle and a dedicated set of artificial lights providing coherent illumination. Additionally, self-prepared algorithms for image acquisition, processing, segmentation, and contour detection were used to analyze wall deformation. Finally, the shape deformation factor was applied to evaluate aorta’s deformation. Experimental results were confronted with medical data from AngioCT and 2D speckle-tracking echocardiography (2DSTE).

**Results:**

Image square analyses indicated that the optimal distance between the camera’s lens and the investigated object was in the range of 0.30–0.35 m. There was approximately 1.44% difference observed in aneurysm diameters between NIVBS (86.57 ± 5.86 mm) and AngioCT (87.82 ± 6.04 mm) (*p* = 0.7764). The accuracy of developed algorithm for the reconstruction of the AAA deformation was equal to 98.56%. Bland–Altman analysis showed that the difference between clinical data (2DSTE) and predicted wall deformation (NIVBS) for all patients was 0.00 mm (confidence interval equal to 0.12 mm) for aneurysm size, 0.01 mm (confidence interval equal to 0.13 mm) and 0.00 mm (confidence interval equal to 0.09 mm) for the anterior and posterior side, as well as 0.01 mm (confidence interval equal to 0.18 mm) and 0.01 mm (confidence interval equal to 0.11 mm) for the left and right side. The optimal range of camera’s lens did not affect acquired values.

**Conclusions:**

The NIVBS with proposed algorithm that reconstructs the pressure from surrounding organs is appropriate to analyze the AAAs in water environment. Moreover, NIVBS allowed detailed quantitative analysis of aneurysm sac wall deformation.

## Introduction

Aortic aneurysm poses a serious health risk because it weakens the vessel’s wall and significantly changes blood hemodynamics [1–3]. The number of patients with diagnosed abdominal aortic aneurysm (AAA) is still increasing, concerning 5% of patients over 65 years of age. The AAA treatment depends on its diameter. When it is lower than 40 mm pharmacological treatment is applied, while the AAA with diameter equal or above 55 mm or growth rate over 5 mm every 6 months requires surgical repair either open or endovascular [4–6].

In clinical diagnostics, combination of different techniques, e.g., echocardiography [7], 3D ultrasound [8], 4D ultrasound strain (4D-US) [9], ultrasonography [10], or computational fluid dynamics (CFD) [11–14], is applied to assess spatial configuration of the vessel and its wall deformation [15]. Moreover, ultrasound image technique may be applied for motion tracking of the carotid artery wall [16, 17] or carotid intimal-media border [18]. Also, it was presented that triangulation may be applied for the tracking of acute stress [19], multi-objective workflow may be used for reinforcement learning [20], and manifold-ranking algorithms may be used to retrieving video shots from brain imaging [21]. However, these techniques do not provide information in real time because raw data collection requires post-processing with dedicated software. When average growth of aneurysm diameter is approximately 5 mm per year, this indicates frequent diagnosis of its condition [22]. On the contrary to in vivo examination, there are ex vivo approaches basing on artificial models of an AAA, e.g., investigation of silicon model of an AAA in a pulsatile artificial circulatory system [10, 23]. Such approach enables application of optical methods supporting or even replacing ultrasound examination. One of the non-contact optical methods applied in image processing is Digital Image Correlation (DIC) used for the measurement of changes in analyzed images [24, 25]. This technique may be used for determination of object deformation [26–28], object displacement [29–32], and object strain [33, 34]. Due to the recent development and increasing accessibility of image acquisition systems and decreasing costs of their production, potential use of DIC technique is still growing, e.g., in mechanical testing applications [35–37], biology [38, 39], or art [40]. So far, few efforts have been taken to exploit the potential of DIC in estimation of spatial configuration of the AAA and deformation of its wall. As quantitative evaluation of such deformation is crucial in assessing wall rupture, the objective of this work is presentation of Non-Invasive Vision-Based System (NIVBS) for the analysis of 3D elastic artificial abdominal aortic models for different physiological and pathological conditions. In the section II medical data, measurement set-up, camera calibration, image analysis, system verification, and statistical methods applied in the paper are described. Section III presents the results directed in the mathematical description of aortic wall deformation with the use of NIVBS. In the section IV, proposed system properties are discussed, while section V concludes the paper.

## Materials and methods

### Medical data

**Fig. 1:**
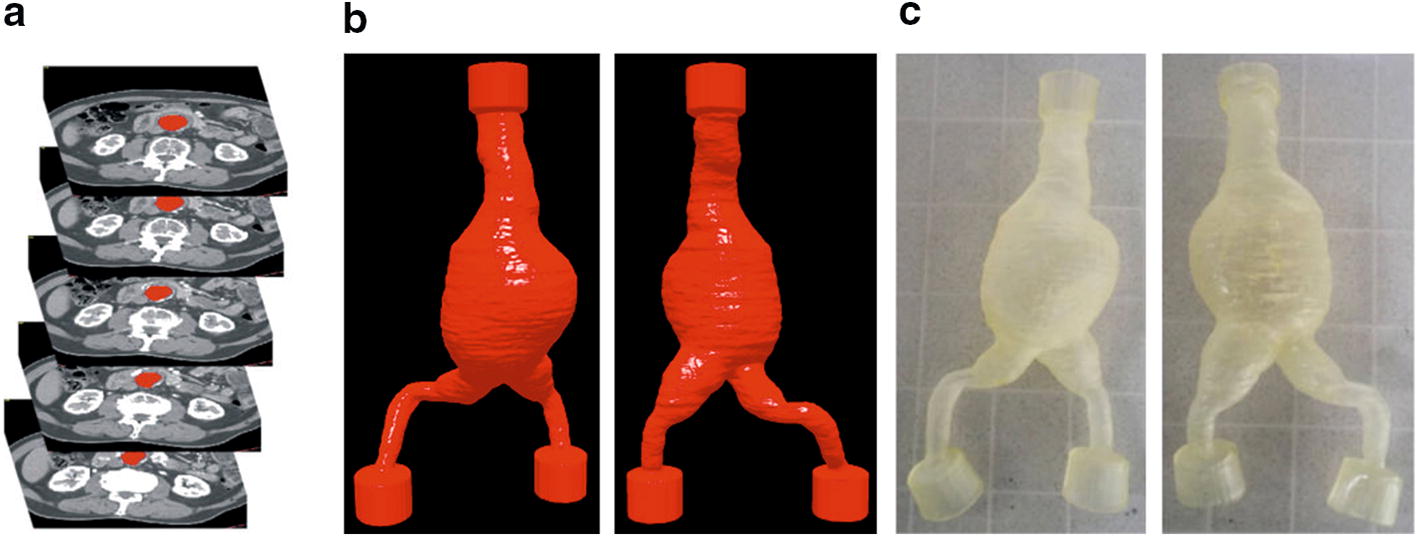
An example of 3D reconstruction of abdominal aortic aneurysm (AAA). **a** AngioCT data—AAA cross sections of abdominal aorta, **b** 3D virtual models reconstructed from AngioCT data (front and back side of an abdominal aortic aneurysm); **c** 3D elastic models printed with the use of 3D printer (front and back side of an abdominal aortic aneurysm)

3D geometries of the AAA were reconstructed with the use of medical data obtained from AngioCT examination (Fig. 1a) (GE Light-Speed 64 VCT; GE Healthcare, Fairfield, CT, USA) of four male patients and 3DDoctor software (Able Software Corp., Lexington, MA, USA) [2, 41]. On every DICOM (Digital Imaging and Communications in Medicine) AAA cross-section region of interest was marked (Fig. 1a). Next, 3D reconstruction (Fig. 1b) was performed in such a way that in each case the inlet to the geometry was just below the mesenteric artery outlet and the outlets ended with the iliac arteries [42–44]. Average diameters of analyzed aneurysms were as follows: 95 mm, 89 mm, 84 mm, and 81 mm with average wall thickness equal to 1 mm. Patients’ data were retrospectively collected based on written informed consent. All medical data and images were anonymized by coding information before assessment and analysis [45]. The study protocol was approved by the local ethics committee on Medical University of Lodz (RNN/126/07/KE).

3D elastic models of aorta (Fig. 1c) were prepared with the use of 3D printing technique (3D printer, Object Eden 350, USA) and Tango Plus material with the following parameters: tensile strength 0.8–1.5 MPa, elongation at break 170–220%, compressive set 4–5%, shore hardness 26–28 scale A, tensile tear resistance 2–4 kg/cm, polymerized density 1.12–1.13 g/cm^3^.

For real hemodynamic reconstruction, the electrocardiography (ECG) traces from four patients were analyzed and the injection volume and frequency of pulsation were defined [46]. Those parameters were used as inlet boundary conditions for particular analysis. To standardize measurements, we used injection volume equal to 70 ml and the frequency of pulsation equals 72 min^−1^ [43, 47]. Additionally these parameters were used as inlet boundary conditions for the dedicated electric impulse generator combined with pulsating pump localized above elastic models. This approach was similar to the one described by Deplano et al. [48]. Moreover, aneurysm wall deformation for each patient was defined with the use of 2D speckle-tracking echocardiography (2DSTE) [49]. Depending on the patient, the average wall deformation was approximately 4.37 mm, 3.97 mm, 3.68 mm, and 3.53 mm.

### Measurement set-up

**Fig. 2:**
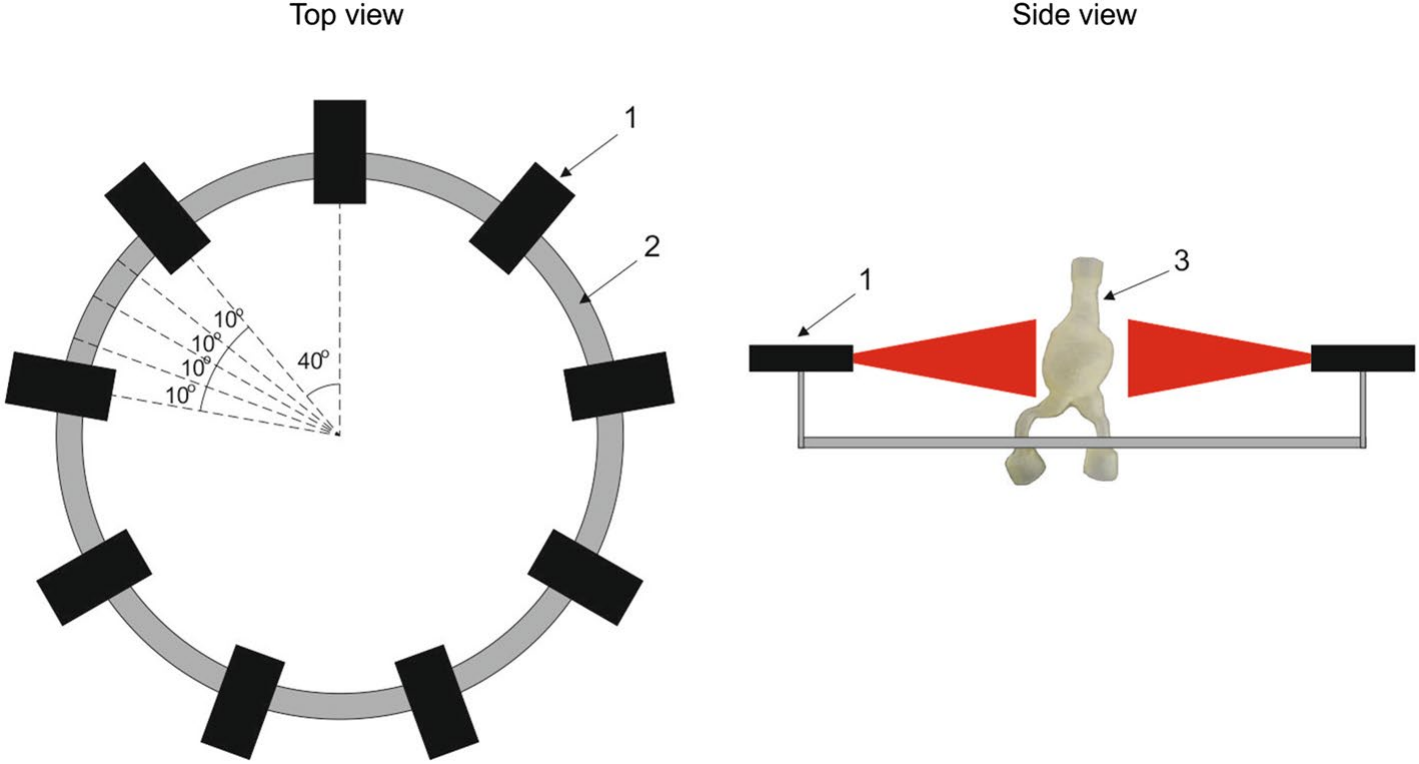
A schema of a Non-Invasive-Vision-Based System NIVBS composed of the following parts: 1—a camera (nine altogether); 2—an aluminum circular profile; 3—an analyzed artificial elastic model of the AAA

The developed NIVBS system consists of nine cameras of the same type (Full HD, resolution: 1920 × 1080, frame rate: 30 frames per second), combined in one set to analyze 3D models of AAA for different sizes of aneurysm sac (Fig. 2).

Cameras were placed on a dedicated circular construction that allowed maintenance of constant distance between each two cameras and between the camera’s lens and the visualized object. The distance between each two cameras was equal to 0.70 rad. (40 deg.), and the distance from the camera’s lens to the middle point of the container with AAA model was set experimentally.

A dedicated set of artificial lights (LED technique) providing coherent illumination during data recording was used. This approach has reduced the impact of changing daylight on data acquisition. Moreover, a set of cameras was placed in the parallel position to the floor to provide capability of horizontal movement. To increase cameras’ accuracy, they could move with 0.17 rad. (10 deg.) step in both sides in the range of 0.70 rad. (40 deg.).

A set of cameras was combined with portable workstation Dell Precision M6400 equipped with four core Intel CPU (2.4 GHz), 4 GB RAM (1333 MHz), and 500 GB SSD HD. To evaluate the AAA deformation, a set of images containing 10 cycles of contraction and relaxation of reprinted 3D physical AAA model were recorded, for 36 angular camera positions.

### Image analysis

**Fig. 3:**
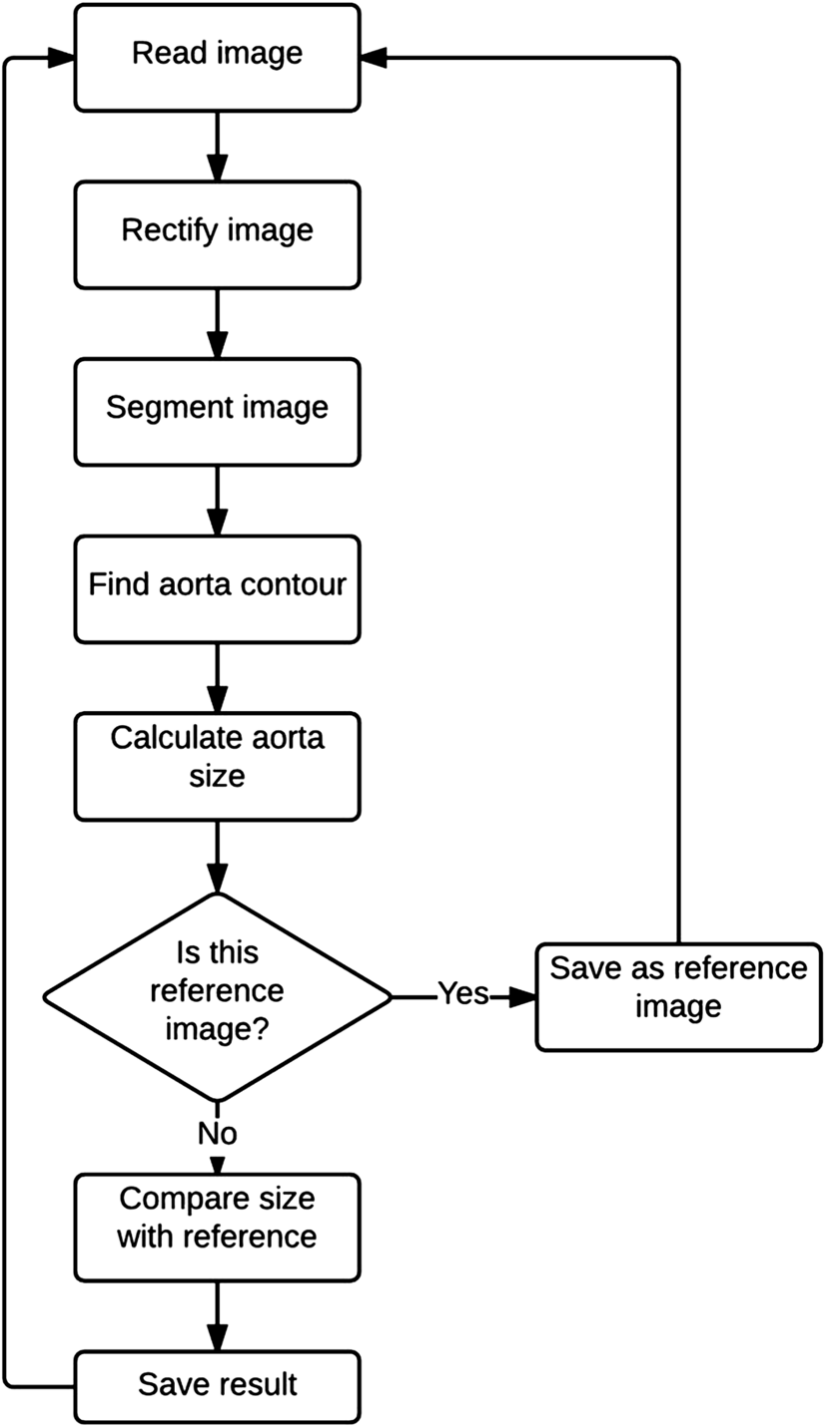
Flowchart of image analysis algorithm

Finally, image analysis algorithm for assessment of the wall deformation the AAA was applied. As the developed algorithm is time-consuming (approximately 0.5 s per image), the collected images were analyzed offline (after acquisition of all images). Four-step image analysis was performed: (1) image rectification, (2) image segmentation, (3) contour detection, (4) evaluation of the aorta deformation (Fig. 3).

#### Image processing

This step was performed to remove barrel, pincushion, or tangential distortions that affected the images during its acquisition. For this purpose, the calibration of the set of cameras was performed within the NIVBS. The mathematical algorithm [50] prepared for camera calibration enabled estimation of matrix of coefficients describing cameras’ physical parameters (e.g., camera’s focal length and image center) and distortions [51].

**Fig. 4:**
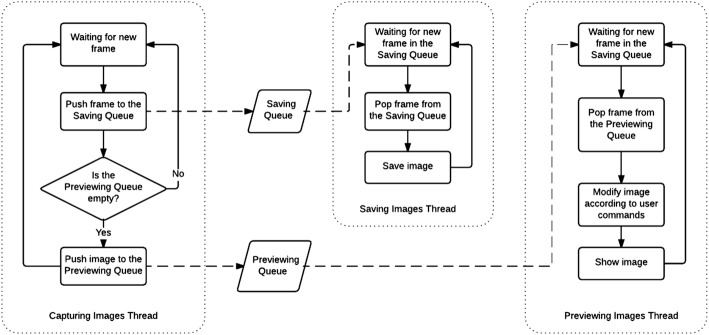
Blok diagram of image acquisition algorithm

The mathematical algorithm for image acquisition was prepared using the “producer–consumer” procedure. The architecture of procedure was as follows: (1) Capturing Images Thread (producer thread), (2) Saving Images Thread (the first consumer thread), (3) Previewing Images Thread (the second consumer thread) (Fig. 4). The Capturing Images Thread (1) was responsible for collecting frames acquired by the cameras and assigning them into two queues (saving queue and previewing queue) allocated in the memory. The first queue was consumed by the Saving Images Thread (2). The second queue was consumed by the Previewing Images Thread (3). As the Capturing Images Thread had the highest priority (it was obligatory for the operating system to allow this thread to work whenever it needs), it could combine all the images produced by the cameras with their highest frame rate. Moreover, the Capturing Images Thread did not consume much CPU. Therefore, it only forwarded the frames from cameras to the queues and then was suspended until another frame appears.

The Saving Images Thread (2) was responsible for popping frames from the saving queue to allocate them on the hard disc. However, the performance of the hard disc was unpredictable and it was obligatory to save all frames immediately when the Saving Images Thread got access. This indicated to collect all frames in the queue limited only by the maximum size of the computer memory avoiding the situation when only one frame per second (or even less) would be saved instead of 30 frames per second necessary for the proper analysis. The priority level for the Saving Images Thread was one step lower compare to the Capturing Images Thread.

The Previewing Images Thread (3) was responsible for both, showing collected images and interaction with the user. The size of a queue in this thread was limited to one image. It was necessary to avoid the situation when the user could select different view modes and affect a lot of CPU consumption. This operation as a consequence could pop frames from the preview queue slower than the Capturing Images Thread would be able to assign them to the queue. This would affect that the user would see images with a few seconds delay. Moreover, there appears no need for the user to check all the collected frames by the camera in the real time. The main point is to check the current image. Therefore, a low frame rate of showing images did not affect any issues.

Moreover, there appeared no dangers for the one image to be pushed by another image in the Capturing Images Thread, unless the Previewing Images Thread would take the image from the previewing queue to process it and show it to the user. Capturing Images Thread ignores pushing new images into previewing queue, unless the Previewing Images Thread would take the image from the previewing queue to process it and show it to the user. As the Previewing Images Thread had the highest CPU consumption, it had assigned the lowest priority.

**Fig. 5:**
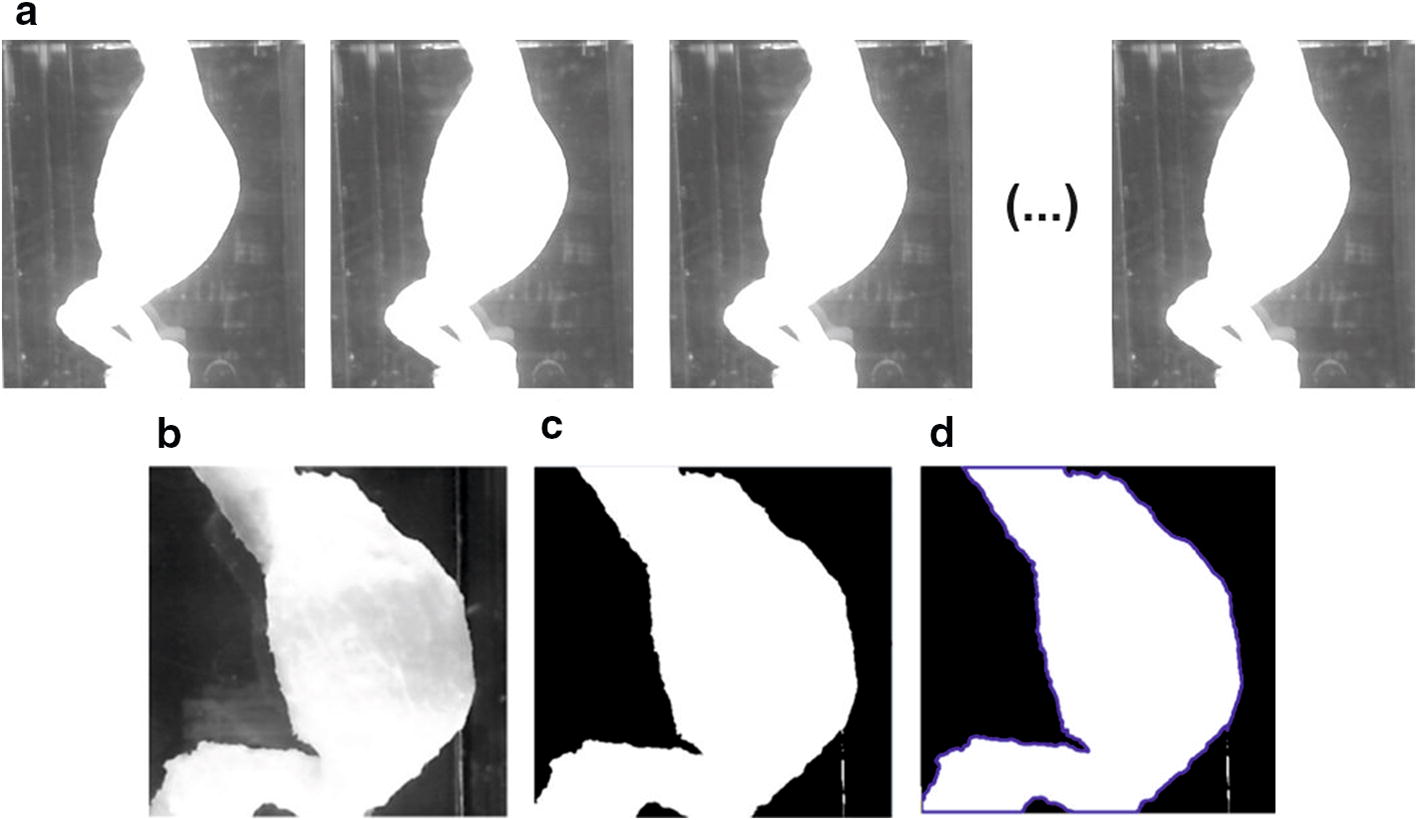
Sample of image analysis for 3D model of AAA: **a** captured image of the 3D model of the AAA; **b** rectification result; **c** threshold segmentation using Otsu algorithm; **d** contour detection of the biggest image object

In the next step, such matrix was applied in the image analysis stage to undistorted images data captured with set of cameras during acquisition stage [52, 53]. A Pinhole camera model was applied to describe the relation in the coordinate system between object points of physical 3D geometry of the AAA and their projection into the image plane [54, 55]. A linear projection of 3D geometry of AAA points into the image plane was assumed. Implemented solution eliminated radial and tangential distortion [56]. Sample acquired image and result of geometrical distortions elimination are shown in Fig. 5a, b, respectively.

#### Image segmentation and contour detection

An automatic image segmentation was applied using Otsu algorithm [57] (Fig. 5c). This method assumes that the image contains two groups of pixels following bi-modal histogram (foreground pixels that correspond to AAA and background pixels). Then the optimum threshold is estimated to separate the two groups to assure minimal variance of brightness distribution for each pixel group. The segmentation was performed based on the following assumptions: (1) the image was converted to the gray-level space, (2) image of aorta is bright, and (3) the rest of the image represents dark background. Maintaining appropriate lighting conditions during acquisition satisfies assumptions (2–3); moreover, it is possible to obtain correct AAA segmentation with the use of the Otsu-based thresholding approach. In the resulting binary image, the aorta was represented with the value 1 while the background with value 0, respectively, as shown in Fig. 4c. Aorta size was estimated by evaluating the area of the biggest object detected in the segmented image, represented by its contour (Fig. 5d).

#### Evaluation of the aorta deformation

Finally, for the estimation of aorta’s deformation the shape deformation factor (Eq. 1) was applied.

1 $$n_{i} = F_{ic} /F_{c} ,$$

where *F*_*ic*_ is the field size in the i-th image from *c*-th camera; *i* the number of the analyzed image from the acquired sequence, c the camera number, *c*∈ < 1,…,9 > , and *Fc* is the field size in the reference image from *c*-th camera.

Each estimated aorta size was compared to the size of the reference image to calculate the shape deformation factor. The reference image was defined as the first acquired image in the sequence, performing motionless aorta for given camera’s angles. Moreover, the evaluated ratio of wall deformation was stored in the summary output file.

### System verification

**Fig. 6:**
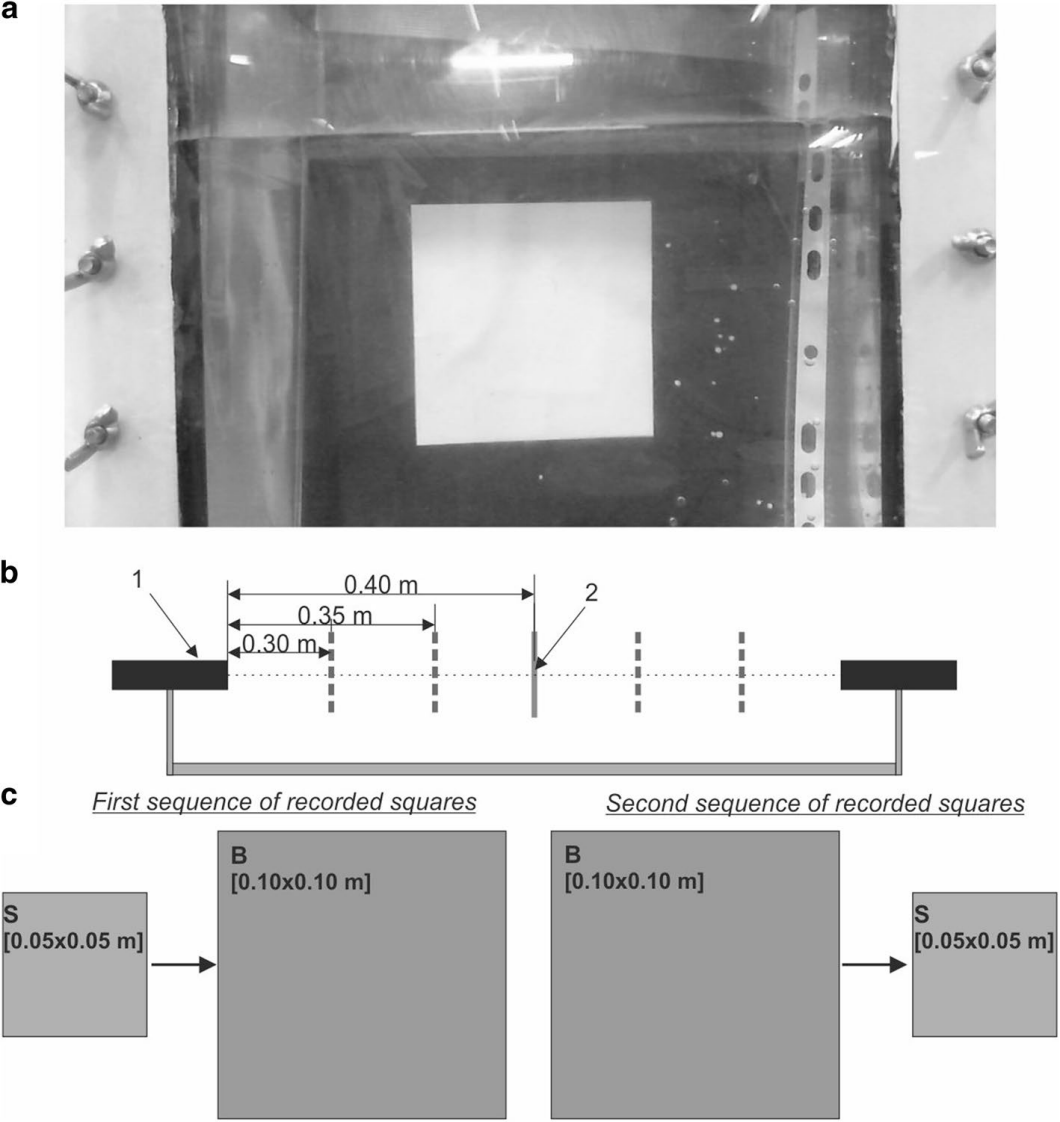
A schema of squares analysis: 1—a camera; 2—analyzed square; **a** a square placed in a container for further AAA models analysis; **b** spatial locations of the analyzed square, considering different distances between the square and camera lens: 0.30, 0.35, and 0.40 m; **c** the investigated sequences of two image squares: first sequence representing small and big square image, second sequence containing the same images acquired in the opposite order; (S: small square: 0.05 × 0.05 m and B: big square: 0.10 × 0.10 m)

Before measurement of wall deformation for 3D elastic models of AAA, an accuracy verification of the NIVBS was performed. For this purpose, two 2D squares of different sizes with specified dimensions (0.05 × 0.05 m and 0.10 × 0.10 m) (Fig. 6a) were applied.

Squares were placed one by one inside the container, perpendicular to the axis of the one selected camera, as shown in Fig. 6c. Since the spatial configuration of AAA is not symmetric and the distance of each point of AAA’s wall to the container’s wall varies, the tests were also performed for different positions of squares. Thus, image squares were acquired for three different distances between the square and camera lens of 0.40 m, 0.35 m, and 0.30 m (Fig. 6b). Next, after images analysis described in subsection Image analysis, the deformation factor was calculated as a ratio of estimated areas of the bigger and smaller square. Considering squares sizes, the true value of this ratio is equal to 4, and thus such value was used as a reference when compared with deformation ratios estimated from acquired square images. Since in the further analysis the AAA models were placed in a container filled with different amounts of distilled water (the presence of water enables reconstruction of physiological pressure value inside abdominal part of human body), tests with squares were performed both for the empty and filled container. Presented procedure of system verification allowed estimation of the average deformation ratio introduced by the acquisition system (a set of cameras) and thus evaluated the system accuracy of the obtained deformation range for the irregular aneurysm sac wall.

Finally, data from AngioCT were used to verify the accuracy of the NIVBS. For each 3D elastic model of AAA, an average diameter of an aneurysm sac without any flow was calculated with the use of NIVBS and compared to the diameter computed from AngioCT data. Moreover, data from 2DSTE were used to verify the accuracy of wall deformation recorded by NIVBS.

### Statistical analysis

Statistical analysis was performed using Statistica 12.0 (Statsoft). Data were presented as mean ± SD (standard deviation). Moreover, Bland–Altman method [58] was applied to analyze the agreement between AngioCT (Computed Tomography Angiography) and NIVBS data as well as between 2DSTE and NIVBS. Spearman’s correlation analysis was used in addition. Comparisons between groups were performed using Student’s t test after verifying normality and variance. Data were considered as significantly different when *p* < 0.05 unless otherwise noted.

## Results

Each time AAA models were placed inside the container filled with water to mimic the influence of organs’ tension on the AAA. Different pressures acting on the AAA models were controlled with the level of water inside the container. Therefore, at the beginning no influence of water amount on acquisition of wall deformation by the NIVBS was analyzed. A verification of the NIVBS for 2640 examinations conducted for two different size squares (smaller—0.05 × 0.05 m and bigger—0.10 × 0.10 m) was performed. Different distances from the camera’s lens to the object (0.40 m, 0.35 m, 0.30 m) placed in the empty and filled container with water were tested. There was no significant difference for both analyzed squares placed in a container with or without water for the distance 0.30 m and 0.35 m (Table 1). While for the distance 0.40 m for the bigger square there was significant difference between the deformation factors 1.00164 ± 0.00018 and 1.00170 ± 0.00016, in water and without water, respectively (*p* = 0.0084).

**Table 1:** Changes in deformation factor *n*_*i*_ for the analyzed squares (smaller—0.05 m × 0.05 m and bigger—0.10 m × 0.10 m) for different environments (with and without water) and different distances (0.40 m, 0.35 m, 0.30 m) between the camera’s lens and analyze squares

Object	Environment	Distance [m]	Average deformation factor *n*_*i*_ [-]	P
Square 0.05 × 0.05 m	Without water	0.30	1.00153 ± 0.00022	0.1837
	With water		1.00150 ± 0.00022	
	Without water	0.35	1.00160 ± 0.00021	0.5499
	With water		1.00159 ± 0.00017	
	Without water	0.40	1.00166 ± 0.00017	0.1020
	With water		1.00169 ± 0.00015	
Square 0.10 × 0.10 m	Without water	0.30	1.00149 ± 0.00023	0.7750
	With water		1.00149 ± 0.00023	
	Without water	0.35	1.00155 ± 0.00020	0.1931
	With water		1.00158 ± 0.00019	
	Without water	0.40	1.00164 ± 0.00018	0.0084
	With water		1.00170 ± 0.00016	

For each case, *n* = 220 analyses were performed. *P* values were calculated using Student’s t test

Next, to simulate the effect of moving irregular AAA wall the sequence of smaller and bigger squares placed one after another inside the container with and without water inside was analyzed. There was no significant difference for deformation factor for distance 0.30 m (4.03018 ± 0.00053 and 4.03016 ± 0.00054 for water and without water, respectively) (*p* = 0.6615). Similar observation was for the distances 0.35 m and 0.40 m (Table 2). According to the results, the accuracy of the NIVBS was about 99.84%.

**Table 2:** Changes in deformation factor *n*_*i*_ for the sequence of smaller and bigger squares placed one after another inside the container (with and without water) and different distances (0.40 m, 0.35 m, 0.30 m) between the camera’s lens and analyzed squares

Object	Environment	Distance [m]	Average deformation factor *n*_*i*_ [-]	*P*
Square 0.05 × 0.05 m → square 0.10 × 0.10 m	Without water	0.30	4.03018 ± 0.00053	0.6615
	With water		4.03016 ± 0.00054	
	Without water	0.35	4.03016 ± 0.00053	0.4856
	With water		4.03019 ± 0.00052	
	Without water	0.40	4.03021 ± 0.00083	0.5630
	With water		4.03016 ± 0.00071	

For each case, *n* = 220 analyses were performed. *P* values were calculated using Student’s t test

Image square analyses indicated that the optimal distance between the camera’s lens and the investigated object with and without water inside the container was in the range of 0.30–0.35 m. Therefore, further analyses of wall deformation of AAA models placed in water for the distance 0.35 m were performed. Firstly, an average aneurysm diameter for each 3D model without application of flow was calculated with the use of NIVBS. The results were compared to the average diameters of aneurysms computed with the use of AngioCT data (Table 3). There was approximately 1.44% difference observed in aneurysm diameters between NIVBS (86.57 ± 5.86 mm) and AngioCT (87.82 ± 6.04 mm) (*p* = 0.7764). The highest difference was observed for the patient P2 (2.05%), while the lowest for the patient P3 (0.84%).

**Table 3:** Aneurysm diameter calculated with the use of NIVBS and AngioCT

Patients	Diameter [mm]		*P*
	NIVBS	AngioCT	
P1–P4	80.50–94.10	81.70–95.40	0.7764

The number of patients *n* = 4. Data are presented as range (min–max) from four patients. *P* value was calculated using Student’s t test

**Fig. 7:**
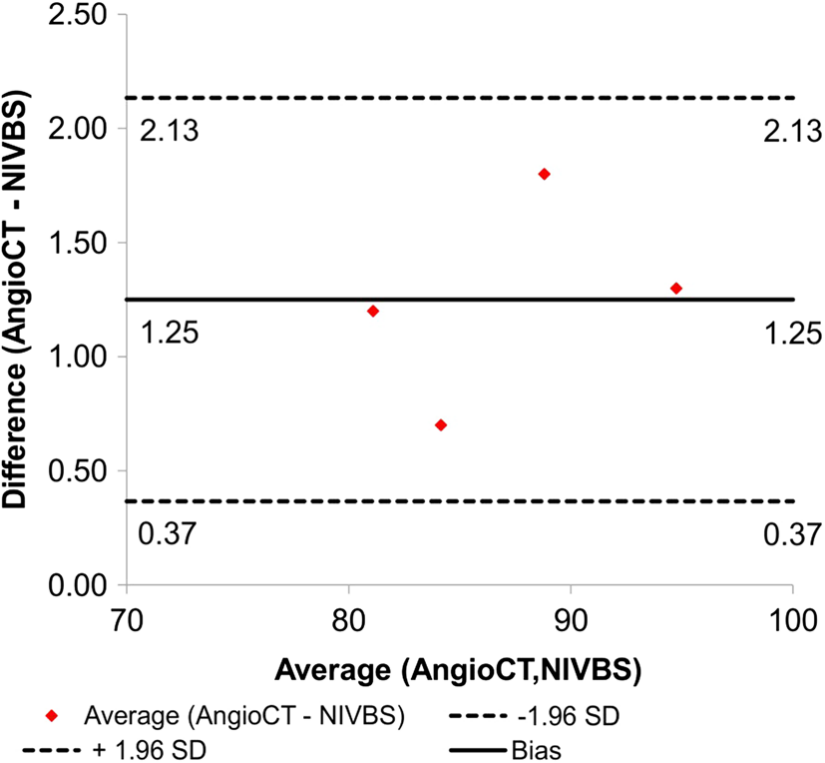
Comparison of the NIVBS and AngioCT for 3D models of AAA with the use of Bland–Altman analysis

Results indicated the accuracy of the NIVBS equal to 98.56%. Moreover, according to Bland–Altman analysis for the AAA models the difference for acquired diameter between the NIVBS and AngioCT was equal to 1.25 mm for the range equal to 2.50 mm (Fig. 7).

**Fig. 8:**
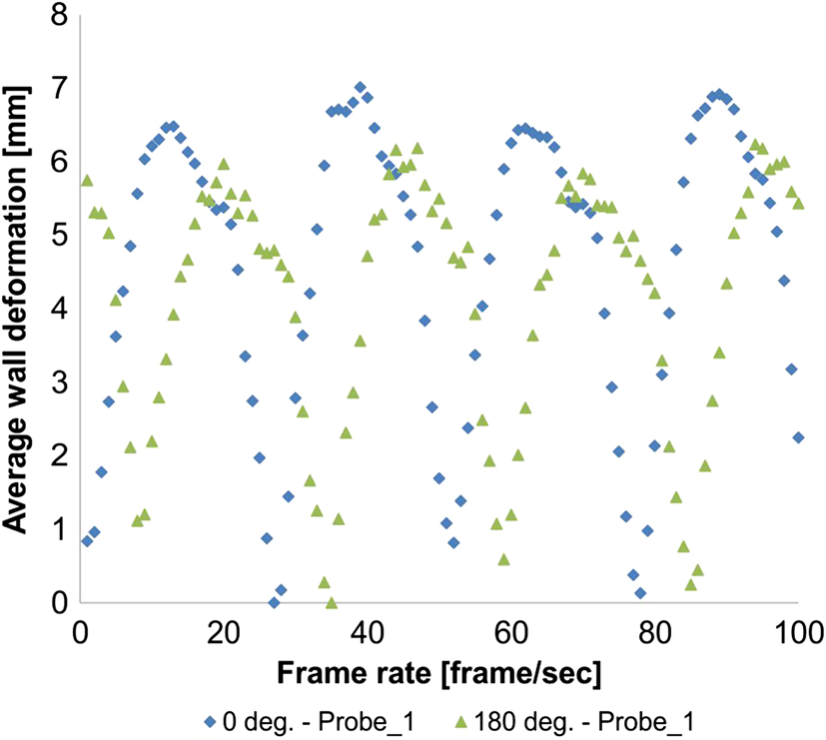
An example of wall deformation in function of frame rate for 1 patient for 2 angles (anterior—0 deg. and posterior—180 deg.)

Next, aneurysm wall deformation for the real hemodynamic for each patient was investigated. The NIVBS could record approximately 30 frames per second (Fig. 8).

NIVBS with the use of nine cameras and dedicated algorithm could reconstruct three-dimensional wall deformation for 3D models of the AAA. Irregular shape of analyzed models was captured by 9 cameras. Depending on the patient, analyzed angle, and shape of aneurysm, different wall displacements were recorded. With increase of aneurysm diameter, higher wall deformation was observed. Moreover, comparison of NIVBS results with 2DSTE data indicated no significant changes. For the patient with the biggest aneurysm, wall deformation was equal to 4.37 ± 0.54 mm and 4.37 ± 0.55 mm, for NIVBS and 2DSTE, respectively (*p* = 0.9976) (Table 4). However, for the patient with the smallest aneurysm diameter wall deformation was equal to 4.37 ± 0.54 mm and 4.37 ± 0.55 mm, for NIVBS and 2DSTE, respectively (*p* = 0.8754) (Table 4).

**Table 4:** Wall deformation measured for NIVBS and 2DSTE four angels (anterior—0 deg., left—90 deg., posterior—180 deg. and right—270 deg.)

Patients	Position	NIVBS					2DSTT					*P*
		Probe				AWD *n*_*i*_ [mm]	Probe				AWD *n*_*i*_ [mm]	
		1	2	3	4		1	2	3	4		
1	A	4.86	4.67	4.87	4.78	4.37 ± 0.54	4.83	4.82	4.81	4.85	4.37 ± 0.55	0.9976
	P	5.12	4.87	4.91	4.88		4.94	4.97	4.99	5.00		
	L	3.92	3.92	3.73	3.79		3.79	3.84	3.82	3.77		
	R	4.16	3.89	3.96	3.58		3.88	3.91	3.93	3.78		
2	A	4.13	3.99	4.10	4.07	3.97 ± 0.19	4.10	4.06	3.99	4.01	3.95 ± 0.16	0.8905
	P	4.16	4.15	4.21	4.16		4.10	4.15	4.20	4.15		
	L	3.92	3.75	3.58	3.92		3.73	3.76	3.78	3.75		
	R	3.89	3.73	3.79	3.96		3.93	3.81	3.83	3.87		
3	A	3.79	3.54	3.61	3.78	3.68 ± 0.23	3.72	3.65	3.61	3.68	3.67 ± 0.18	0.9730
	P	3.86	4.02	3.98	4.04		3.95	3.99	3.94	3.96		
	L	3.26	3.66	3.74	3.59		3.49	3.54	3.52	3.55		
	R	3.56	3.57	3.40	3.45		3.58	3.56	3.51	3.50		
4	A	3.59	3.52	3.63	3.55	3.53 ± 0.19	3.63	3.59	3.61	3.59	3.55 ± 0.16	0.8754
	P	3.77	3.77	3.77	3.79		3.76	3.81	3.74	3.73		
	L	3.40	3.47	3.51	3.39		3.51	3.47	3.53	3.49		
	R	3.50	3.15	3.43	3.24		3.32	3.31	3.38	3.35		

The number of patients was *n* = 4. Each patient was analyzed in 4 probes. Each probe for NIVBS was composed of 30 samples. A: Anterior, P: posterior, L: left, R: right, Average wall deformation *n*_*i*_—AWD *n*_*i*_

**Fig. 9:**
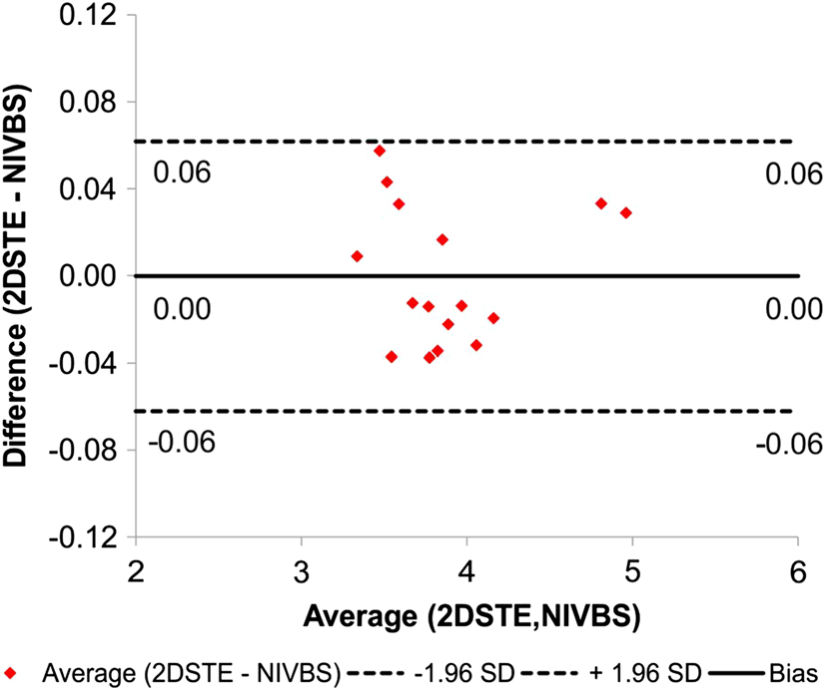
Comparison of the NIVBS and 2DSTE for all AAA models with the use of Bland–Altman analysis

Moreover, according to Bland–Altman analysis the difference between clinical data (2DSTE) and predicted wall deformation (NIVBS) for all 4 patients was 0.00 mm (confidence interval equal to 0.12 mm) (Fig. 9).

**Fig. 10:**
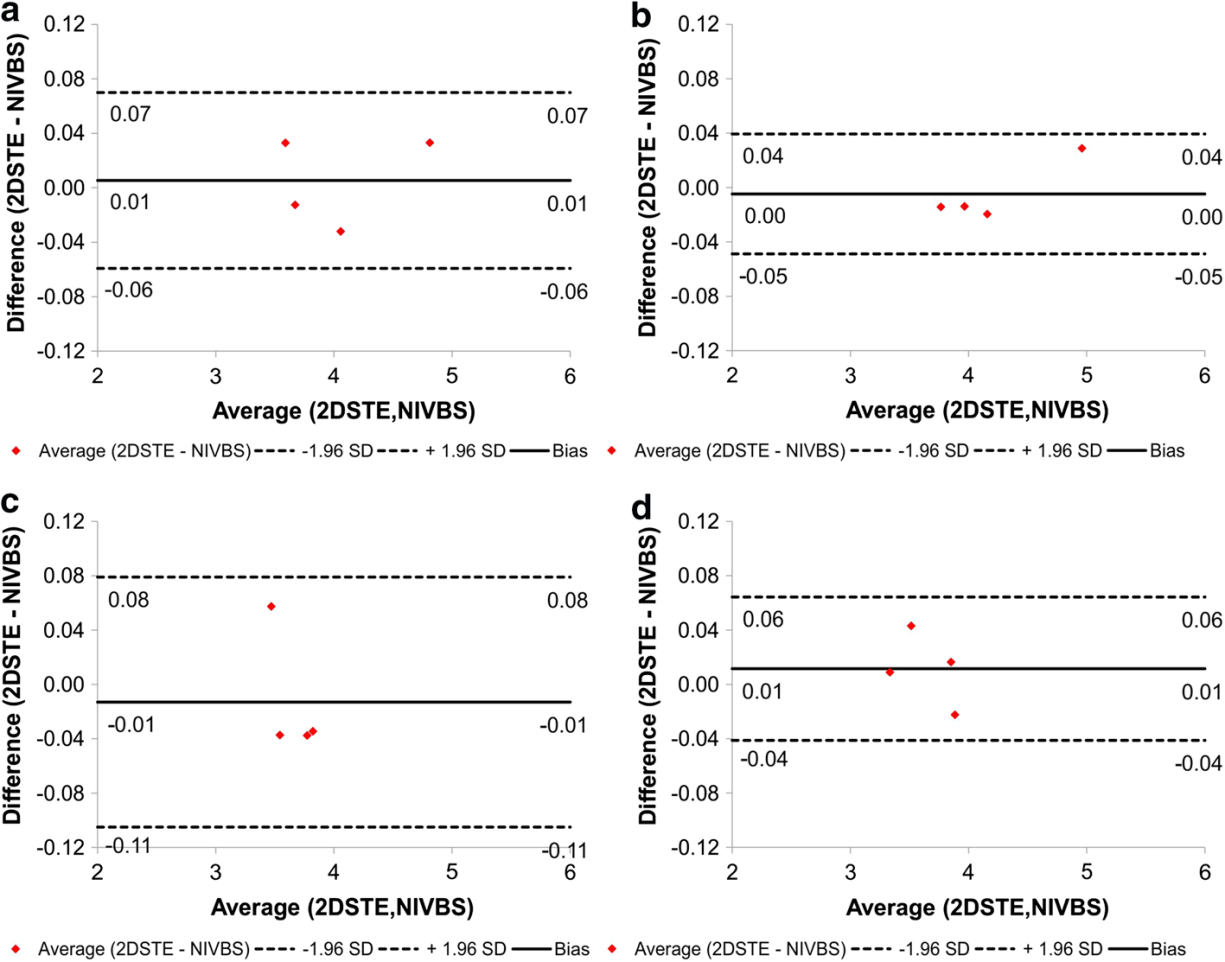
Comparison of the NIVBS and 2DSTE for four sides of AAA model separately with the use of Bland–Altman analysis for **a** anterior; **b** posterior; **c** left; **d** right

Finally, for each AAA model wall deformation was analyzed for anterior, posterior, left, and right side separately. The highest wall deformation was observed for the posterior, while the smallest for the left and right side. Additionally, according to Bland–Altman analysis the difference between clinical data (2DSTE) and predicted wall deformation (NIVBS) for all 4 patients for anterior and posterior was equal to 0.01 mm (confidence interval equal to 0.13 mm) and 0.00 mm (confidence interval equal to 0.09 mm), respectively (Fig. 10a, b). However, for left and right side the difference between clinical data (2DSTE) and predicted wall deformation (NIVBS) for all 4 patients for left and right side was equal to 0.01 mm (confidence interval equal to 0.18 mm) and 0.01 mm (confidence interval equal to 0.11 mm), respectively (Fig. 10c, d).

## Discussion

The presented NIVBS allowed 360^o^ projection of the AAA models reflecting its actual shape and movement in real time for different physiological conditions. Obtained results demonstrated that remodeling of physiological conditions of blood hemodynamic in ex vivo bioengineering reactor required water environment application, to simulate a tension of organs inside the abdominal part of human body. The existing optical methods applied for ex vivo analyses lack monitoring of mechanical properties of vessels under physiological pressure. Moreover, there are no references presenting application of computer vision systems for 3D projection of vessel’s wall behavior after placement in bioengineering reactors with or without water inside.

Bihari et al. [59] used a commercial real-time 3D speckle-tracking ultrasound system with laser scan micrometry and video photogrammetry to explore local displacement and strain parameters of the whole silicon model of abdominal aortic aneurysm. Despite the number of image acquisition techniques, the methodology did not indicate the clinical application. The authors investigated the aorta horizontally which was contrary to our study where aortas were positioned vertically to minimize the risk of inside air deposition in the aneurysm and to adopt the *g*-force to erect position. Moreover, the analyzed aorta was supplied with pump volume of 33.6 ml and 60 min^−1^; contrary to this, NIVBS was implemented for physiological conditions where ECG traces from four patients were analyzed to measure the heart rate, as well as the injected volume was equal to 70 ml and the frequency of pulsation was equal to 72 min^−1^. Next, to identify changes in aneurysm diameter with the use of 3D ultrasound, Bihari et al. used metal screw nuts. It was in the contrary to our NIVBS where no markers are required to identify 3D shape of the aorta and change in its diameter. Furthermore, NIVBS allowed to move cameras’ position to analyze objects of different sizes, while Bihari et al. fixed a camera in one position. Moreover, Bihari et al. did not consider water environment mimicking internal pressure in human body, and their experimental set-up included only water bath with distilled water without possibility of changing amount of liquid. However, in our NIVBS we investigated both cases without and with water inside the container where analyzed aorta was tested. Therefore, we indicated that changing amount of liquid inside the container does not interrupt acquisition of analyzed parameters but it creates additional outside pressure that mimics pressure in the abdominal cavity. Moreover, laser scan micrometer indicated measurement of 4-cm maximum measurement field transversely which was in the contrary to NIVBS where whole 3d projection of investigated aorta was acquired. Finally, the results of Bihari et al. were not verified with medical data, which was contrary to the NIVBS. Therefore, NIVBS provides more detailed information verified with medical data. Hence, the results of Bihari et al. did not reflect the mechanical behavior of human vessels. On the other hand, Karatolios et al. proposed a combination of 3D ultrasound speckle tracking and FEM analysis to measure aortic wall strain [10]. However, for the analysis of wall motion with the use of FEM tool the authors analyzed exported position of vector fields instead of mechanical model of aortic wall preparation. This approach allowed reconstruction of wall motion, while NIVBS can monitor wall motion of analyzed aortic model. Furthermore, Genovese et al. proposed a panoramic digital image correlation method for vascular strain analysis and material characterization [60]. However, the authors dedicated their device to mouse arteries whose anatomical properties differ from the human aorta (length from 4 to 10 mm and inner diameter less than 1 mm). The NIVBS can monitor human aorta (length approximately 300 mm and aneurysm diameter 81–95 mm). Moreover, to create a dark background for the white speckle pattern the authors used Evan’s blue dye. For NIVBS, no markers are required to identify 3D shape of aorta and change in its diameter. Similarly to Karatolios et al., Bersi et al. proposed a novel methodology for characterizing regional variations in the material properties of murine aortas [10, 61]. In their approach, the digital camera was mounted vertically above the murine aortas. This enabled to monitor the motion of straight tube. However, NIVBS can monitor more realistic wall movement representing spatial, irregular shape of aortic aneurysm. Moreover, the proposed device was applied for the analysis of vessels length equal to approximately 7 mm, while NIVBS can monitor vessels with length approximately 300 mm. Additionally, Gulan et al. investigated transparent aortic silicon phantom with the use of 3D particle tracking velocimetry. Their set-up did not include both measurement of spatial configuration as well as water environment surrounding analyzed phantom [62]. Moreover, Rouet et al. [8] combined 3D ultrasound and computer tomography to assess the maximum diameter of patients with AAA. Their approach combined two commercial tools to measure aneurysm diameter. However, they did not consider wall deformation in real time. Fadel et al. [7] proofed that echocardiography is a useful tool for investigation of aortic aneurysms in human organism. Moreover, Metaxa et al. [63] measured aortic aneurysm growth quantification with the use of mathematical algorithm computing aneurysm surface reconstructed from AngioCT data. Their approach strongly depended on optical resolution of AngioCT data. Similarly, Mattes et al. [64] developed an algorithm for the evaluation of follow-up CT scans after endovascular repair. Satriano et al. [65] proposed a 3D image-based approach to compute aortic wall strain maps in vivo. In our study for acquisition aneurysm of sac wall deformation, 3D elastic models of the AAA placed in the transparent container fulfilled with water were used. Pinhole camera model was applied for description of the relation between object points of physical 3D geometry of the AAA and their projection into the image plane. Stein et al. and White et al. investigated Digital Image Correlation (DIC) technique of cross-correlation process to recognize the coefficients of object’s deformation for the description of image patches on two or more corresponding images. However, they assumed that DIC technique was applied for the heterogeneous objects [66, 67]. Contrary to this, the current approach considers the fact that analyzed 3D models of elastic aorta may be characterized by different spatial configurations, while its structure remains homogenous structure. This study indicated that DIC technique will not provide correct results. Therefore, computer vision algorithms for the AAA wall deformation were applied. They enabled separation of aorta’s object from the background and evaluation of deformation ratio in sequence of images captured for hemodynamic conditions reflecting blood flow in analyzed patient.

## Conclusions

In our study, we designed, developed, and verified an optical system for an aneurysm sac wall deformation analysis based on the elastic AAA models reconstructed from AngioCT data. The system was constructed with nine digital cameras and self-made image processing algorithms to investigate wall deformation around the AAA sac. Obtained results confirm that ex vivo approach reflects the AAA wall behavior when compared to the clinical data (USG-Doppler).

As the shape of the aneurysm sac wall was not uniform, different values of its deformation were recorded using different camera angles. Moreover, it was demonstrated that the container with and without water did not affect image acquisition in the NIVBS. Therefore, the AAAs were analyzed only in water environment to reconstruct the pressure from surrounding organs.

The proposed method, combining application of the 3D AAA models and optical methods, allowed detailed analysis of the aneurysm sac wall mechanics. The system accuracy was about 98% and no significant differences between wall deformation acquired with the NIVBS and AngioCT was observed. Application of the presented system may be useful to improve treatment strategies of patients with the AAA. Potential implications comprise prediction of a risk of aneurysm wall rupture by determination of the weakest area in aortic wall. Moreover, verified ex vivo analyses instead of in vivo tests exclude the risk of patient injury or death, providing the same time quantitative and reliable results.

## Limitation to the study

Although our system has some advantages compared to the existed solutions discussed above, we may see some limitations. First, we tested our system in water environment; however, we did not study how liquids with different viscosities may influence wall displacement analysis. Secondly, in this study we tested only abdominal aortic aneurysms only from four patients and further studies on larger group representing different spatial configurations are needed. Finally, we tested aneurysms whose diameter was in the range of 80–90 mm. Those aneurysms are relatively big and studies on smaller cases, e.g., 55–70 mm should be made in the future.

## Data Availability

Not applicable.

## Abbreviations

2DSTE: 2D speckle-tracking echocardiography
A: anterior
AAA: abdominal aortic aneurysm
AngioCT: Computed Tomography Angiography
AWD *n*_*i*_,: average wall deformation *n*_*i*_
DICOM: Digital Imaging and Communications in Medicine
ECG: electrocardiography
F_ic_: is the field size in the i-th image from c-th camera
i: the number of the analyzed image from the acquired sequence
c: the camera number, c∈ < 1,…,9>
Fc: is the field size in the reference image from c-th camera
n_i_: aorta’s shape deformation factor
NIVBS: Non-Invasive Vision-Based System
P: posterior
L: left
R: right
